# Circulating miRNA repertoire as a biomarker of metabolic and reproductive states in rainbow trout

**DOI:** 10.1186/s12915-021-01163-5

**Published:** 2021-11-16

**Authors:** Emilie Cardona, Cervin Guyomar, Thomas Desvignes, Jérôme Montfort, Samia Guendouz, John H. Postlethwait, Sandrine Skiba-Cassy, Julien Bobe

**Affiliations:** 1 INRAE, LPGP, Fish Physiology and Genomics, F-35000 Rennes, France; 2 INRAE, Univ. Pau & Pays Adour, E2S UPPA, NUMEA, 64310 Saint-Pée-sur-Nivelle, France; 3 GenPhySE, University of Toulouse, INRAE, ENVT, F-31326 Castanet-Tolosan, France; 4 Institute of Neurosciences, University of Oregon, Eugene, OR 97403 USA; 5 Institute of Functional Genomics, MGX, UMR 5203 CNRS – U1191 INSERM, F-34094 Montpellier, France

**Keywords:** Biomarker, Biological fluid, Non-invasive phenotyping, *mir202*, myomiR, *mir375*, Fish, The authors Emilie Cardona and Cervin Guyomar contributed equally to this work.

## Abstract

**Background:**

Circulating miRNAs (c-miRNAs) are found in most, if not all, biological fluids and are becoming well-established non-invasive biomarkers of many human pathologies. However, their features in non-pathological contexts and whether their expression profiles reflect normal life history events have received little attention, especially in non-mammalian species. The aim of the present study was to investigate the potential of c-miRNAs to serve as biomarkers of reproductive and metabolic states in fish.

**Results:**

The blood plasma was sampled throughout the reproductive cycle of female rainbow trout subjected to two different feeding regimes that triggered contrasting metabolic states. In addition, ovarian fluid was sampled at ovulation, and all samples were subjected to small RNA-seq analysis, leading to the establishment of a comprehensive miRNA repertoire (i.e., miRNAome) and enabling subsequent comparative analyses to a panel of RNA-seq libraries from a wide variety of tissues and organs. We showed that biological fluid miRNAomes are complex and encompass a high proportion of the overall rainbow trout miRNAome. While sharing a high proportion of common miRNAs, the blood plasma and ovarian fluid miRNAomes exhibited strong fluid-specific signatures. We further revealed that the blood plasma miRNAome significantly changed depending on metabolic and reproductive states. We subsequently identified three evolutionarily conserved muscle-specific miRNAs or myomiRs (miR-1-1/2-3p, miR-133a-1/2-3p, and miR-206-3p) that accumulated in the blood plasma in response to high feeding rates, making these myomiRs strong candidate biomarkers of active myogenesis. We also identified miR-202-5p as a candidate biomarker for reproductive success that could be used to predict ovulation and/or egg quality.

**Conclusions:**

Together, these promising results reveal the high potential of c-miRNAs, including evolutionarily conserved myomiRs, as physiologically relevant biomarker candidates and pave the way for the use of c-miRNAs for non-invasive phenotyping in various fish species.

**Supplementary Information:**

The online version contains supplementary material available at 10.1186/s12915-021-01163-5.

## Background

MicroRNAs (miRNAs) are small non-coding RNAs (about 22nt in length) that act as post-transcriptional gene regulators by inducing mRNA decay or translational repression in animals and plants [1, 2]. In vertebrates, miRNAs are highly conserved and associated with numerous physiological and pathological processes [3]. miRNAs can be secreted from cells into body fluids, such as serum, blood plasma, saliva, colostrum, milk, urine, semen, amniotic fluid, cerebrospinal fluid, peritoneal fluid, and pleural fluid [4–7]. In the past decade, miRNAs have emerged as highly promising biomarker molecules, due to their presence and stability in most biological fluids, including the blood plasma [8]. Circulating miRNAs (c-miRNAs) have thus been documented in many biomedical contexts and have revealed great potential as diagnostic and prognostic non-invasive biomarkers in human medicine for a variety of pathologies, including cancer [9]. In contrast, c-miRNAs in non-pathological contexts have received little attention and data in non-human species remain scarce and for the most part still relate to pathological conditions [10–13]. In a few cases, c-miRNAs have, however, been studied in non-pathological contexts, such as puberty [14], oestrus cycle and pregnancy [15, 16], and embryonic development [17] but also in relation to animal nutrition [18–20] or in response to environmental changes [21–24].

To date, little data exist on c-miRNAs in aquatic species, including in fish, even though the presence of miRNAs was recently reported in the blood plasma of Senegalese sole and rainbow trout [20, 25], in the seminal fluid of Atlantic salmon [26] and in the mucus of rainbow trout [25]. In order to monitor the growth, reproduction, and health of fish stocks in aquaculture or of wild fish populations, current methods necessitate important, repeated, and often stressful manipulations of the animals to collect live measurements. Furthermore, numerous representative specimens often need to be euthanized to for example assess the reproductive state. In contrast, non-invasive sampling of body fluids such as the blood plasma could potentially inform not only of the fish condition but also of its past and present overall growth, reproductive advancement, and health status. Therefore, developing c-miRNAs that could serve as biomarkers of physiological, metabolic, and health status would be of major interest for aquaculture but also for wild population management, including endangered species, for which each wild and captive specimen is critical.

**Fig. 1:**
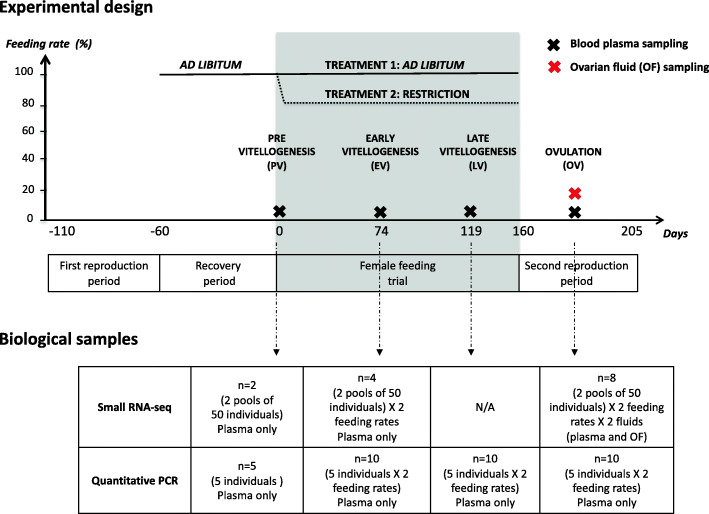
Experimental design. Fish rearing reproductive stages, fluid sampling, and biological samples used for RNA-seq and QPCR analysis are described. Additional details on fish rearing including feeding levels and monitoring of reproduction can be found in [27]

The aim of the present study was thus to investigate the potential of c-miRNAs as biomarkers of reproductive and metabolic states. We therefore aimed at characterizing the rainbow trout c-miRNAome in two biological fluids: the blood plasma and ovarian fluid (i.e., the fluid in which the eggs are bathed after ovulation), which can easily and non-invasively be collected. We investigated two different feeding regimes that trigger contrasting metabolic states [27]. The blood plasma was analyzed at different stages of the reproductive cycle, and ovarian fluid was studied at ovulation as described in Fig. 1. Rainbow trout was chosen due to its relatively large size, which simplifies the fluid collection, its belonging to the economically and culturally important salmonid family, as well as its sequenced genome and available small RNA-seq data in 38 different tissues, cell types, organs, and embryonic stages [28]. In fish, a comprehensive view of the overall miRNA repertoire was only recently characterized [29] but was still lacking in rainbow trout. We thus established a comprehensive annotation of expressed miRNAs in rainbow trout using existing and newly generated sRNA-seq data and using existing miRNAome annotations in teleost fish [30–34]. To gain insight into the organ of origin, diversity, and specificity of c-miRNAomes, blood plasma and ovarian fluid miRNA expression data were analyzed along with data from a panel of 21 tissues and organs. We then evaluated the potential of c-miRNAs to serve as biomarkers of the reproductive stage of female rainbow trout throughout their reproductive cycle. Finally, we evaluated the potential of selected c-miRNAs to serve as biomarkers of the metabolic state through the use of two feeding levels: ad libitum feeding or a physiologically relevant moderate feeding restriction. We used a two-step strategy, relying first on the small RNA sequencing of a limited number of pooled biological samples originating from a large number of individuals to identify major candidate c-miRNA biomarkers and then further evaluating their biomarker potential using quantitative PCR on individual biological replicates and additional reproductive stages. Here, we show that the blood plasma and ovarian fluid miRNAomes are complex and share a majority of common miRNAs. We report, however, strong fluid-specific signatures in terms of expression levels and fluid-specific miRNAs and identified candidate biomarkers of sexual maturation and active myogenesis. Together, these results highlight the relevance and potential of c-miRNAs for non-invasive phenotyping in aquatic species.

## Results and discussion

### The rainbow trout reference miRNAome

In order to evaluate the complexity of the rainbow trout circulating miRNAome, we first established a comprehensive rainbow trout miRNAome that could be used as a reference. We identified and annotated 354 mature rainbow trout miRNAs corresponding to at least 280 miRNA genes by adapting a strategy previously used in different fish species [30, 32] and by using a total of 52 sequencing libraries composed of 14 new libraries (blood plasma and ovarian fluid) and 38 libraries that we had previously been generated in a wide variety of tissues, cellular populations, organs, and whole embryos [28]. The rainbow trout miRNAome was previously incompletely characterized with only 123 mature miRNAs described based on sequence homology with other species [28]. In a recent study, the number of known miRNAs identified in rainbow trout mucus, blood plasma, and surrounding water based on sequence homology with other species ranged from 94 to 192 depending on the samples [25]. With 354 annotated mature miRNAs, the present study therefore corresponds to a major increase in our knowledge of the rainbow trout miRNAome. In addition, existing studies in rainbow trout often rely on miRNA annotations available in other species (e.g., Atlantic salmon) [25] as no rainbow trout miRNA annotation is available in miRBase [35] and other databases [36]. Finally, the rainbow trout miRNAome annotation reported here is consistent with the recently described evolution of miRNA genes in teleost fish [29]. While slightly lower, the number of mature rainbow trout miRNAs reported here is in agreement with previous reports in other ray-finned fish species using similar genome-wide annotation strategies that led to the annotation of 362, 495, 396, and 408 individual mature miRNAs in gar, zebrafish, stickleback, and icefish, respectively [30–32]. In summary, the present report provides a comprehensive and evolutionarily supported rainbow trout miRNA repertoire annotation that was previously incompletely characterized in this species.

### The high complexity of circulating miRNAome

**Fig. 2:**
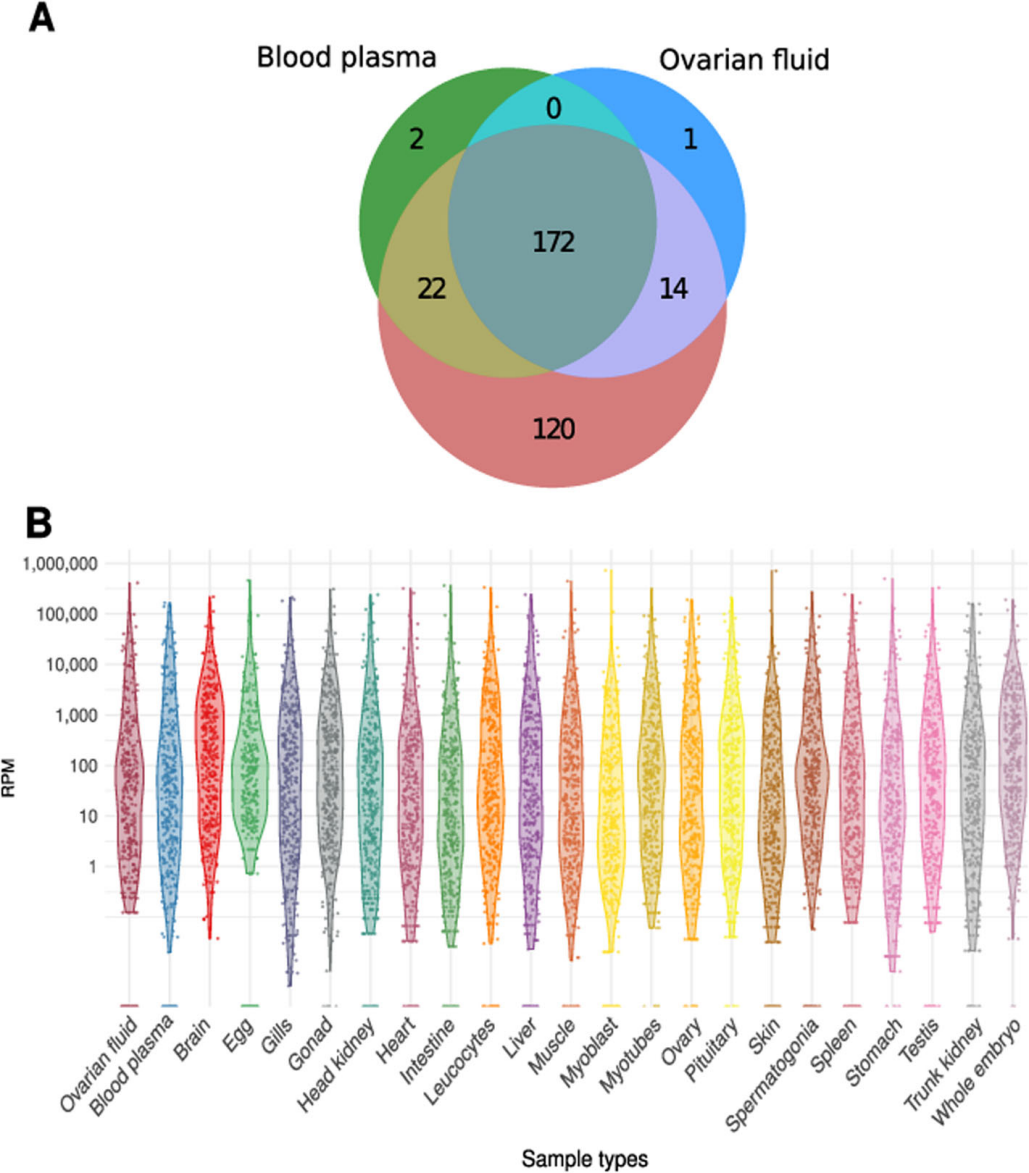
Circulating miRNA repertoires. **A** Venn diagram of miRNAs detected in the blood plasma, ovarian fluid, and in 21 other sample types (brain, eggs, gills, gonad, head-kidney, heart, intestine, leucocytes, liver, muscle, myoblasts, myotubes, ovary, pituitary, skin, spermatogonia, spleen, stomach, testis, trunk-kidney, whole embryos). A miRNA was considered expressed in a sample type when its averaged normalized abundance exceeded 10 RPM (reads per million reads). **B** Distribution of miRNA normalized counts across sample types listed above in RPM. Corresponding data are available in Additional file 4

Among the 354 annotated rainbow trout miRNAs, 331 were detected on average above a threshold of 10 reads per million reads (RPM), either in one of the two biological fluids studied or in one of the 21 other sample types analyzed (brain, pituitary, gills, heart, muscle, myoblasts, myotubes, stomach, intestine, liver, spleen, head-kidney, leucocytes, trunk-kidney, skin, gonad, testis, spermatogonia, ovary, eggs, whole embryos). In biological fluids, 211 miRNAs were identified and corresponded to 64% of the overall expressed miRNAome diversity (Fig. 2A). Among these 211 miRNAs, 172 (82%) were detected in both blood plasma and ovarian fluid, while 24 (11%) were detected only in the blood plasma and 15 (7%) were detected only in the ovarian fluid (Fig. 2A). Notably, two miRNAs (miR-365-2-5p, miR-23b-2-5p) were detected above the 10 RPM threshold only in the blood plasma and not in any other studied sample, while one miRNA (miR-726-5p) was found only in the ovarian fluid (Fig. 2A). A comprehensive analysis of miRNA expression levels revealed that the overall distribution patterns of miRNA read counts were similar in biological fluids and in other samples (Fig. 2B). In each analyzed library, including ovarian fluid and blood plasma libraries, a few miRNAs accounted for most of the reads per million. Together, these data illustrate the relatively large complexity of c-miRNAomes of the two biological fluids studied here. Our results are consistent with existing data in the human, chicken, and cow blood plasma in which 349, 649, and 468 miRNAs were reported, respectively [4, 21, 24]. This result in trout, however, is to our knowledge the first comprehensive characterization of blood plasma and ovarian fluid miRNAomes in fish.

### Origin and specificity of c-miRNAs in the blood plasma and ovarian fluid

**Fig. 3:**
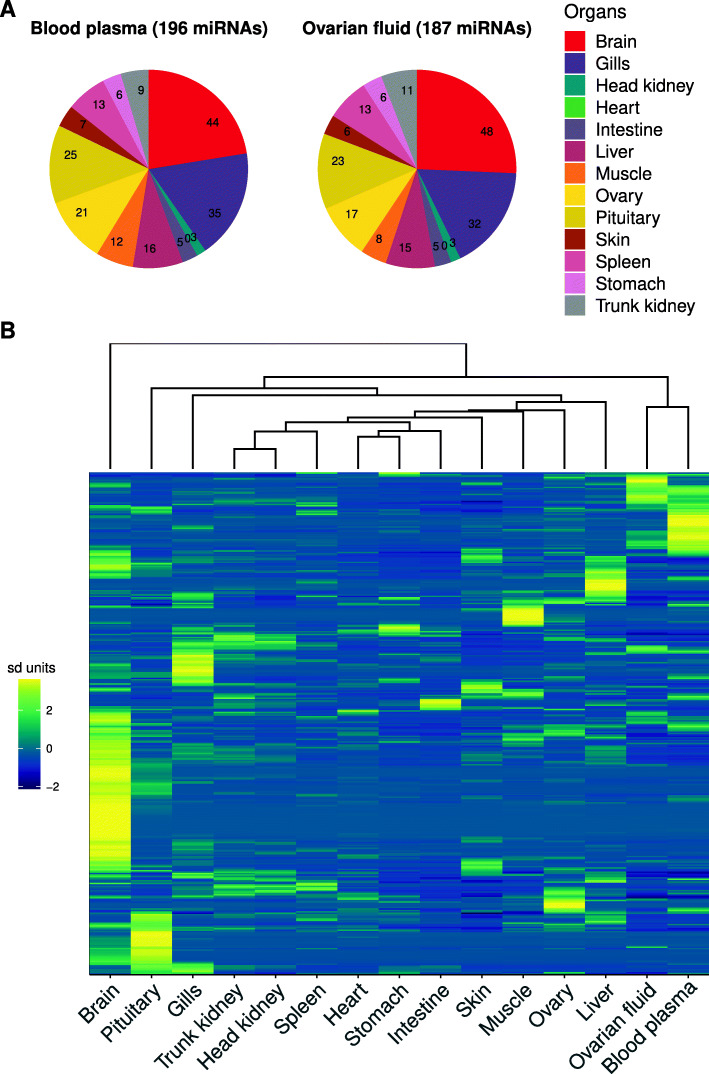
Putative origin and expression patterns of c-miRNAs. **A** For each fluid c-miRNA, the organ in which its expression was the highest was considered as the putative organ of origin. The number of c-miRNAs exhibiting highest expression in a specific organ are shown on the chart. The heart is displayed even though no c-miRNA had a maximum expression in this organ. **B** Heatmap of miRNA expression across different organs, blood plasma, and ovarian fluid. The heatmap was built using a row-scaled matrix of the normalized read counts (RPM) supplied by Prost!. Normalized RPM counts were averaged for all samples of a given sample type. Corresponding data are available in Additional file 1

To investigate the possible organs of origin of miRNAs present in the blood plasma and ovarian fluid, we categorized c-miRNAs based on the organ in which they exhibited the highest expression, under the hypothesis that an organ strongly expressing a miRNA is likely an organ secreting the miRNA into the fluid, or at least one of the major contributors. This analysis investigated a subset of 13 different organs from females (brain, pituitary, gills, heart, muscle, stomach, intestine, liver, spleen, head-kidney, trunk-kidney, skin, ovary) and excluded male samples, complex libraries (e.g., whole embryos), and individual cell types (e.g., myoblasts). We observed that miRNAs present in both ovarian fluid and blood plasma had maximum expression (i.e., were detected at the highest level) in a wide diversity of organs (Fig. 3A). For both analyzed fluids, the brain, gills, pituitary, ovary, and liver were the organs in which most miRNAs were the most highly expressed and no major differences in potential organs of origin could be identified between these two fluids (Fig. 3A). These data suggest that many organs might contribute to the complexity of c-miRNAomes in both blood plasma and ovarian fluid. It is however noteworthy that the different organs used in the analysis likely contained some blood at the time of sampling. This could have led to an overestimation of the possible organs of origin of specific c-miRNAs, especially for the organs exhibiting low expression of these c-miRNAs. In addition, a recent study in rainbow trout showed that the abundance of specific miRNAs following a stressful event exhibited an inverse relationship between tissues and blood plasma extracellular vesicles that could indicate that the liver and head kidney secreted these miRNAs [37]. Further analyses monitoring miRNA abundance in fluids and putative tissues and organs of origin over time are needed to further understand the origin of c-miRNAs.

**Fig. 4:**
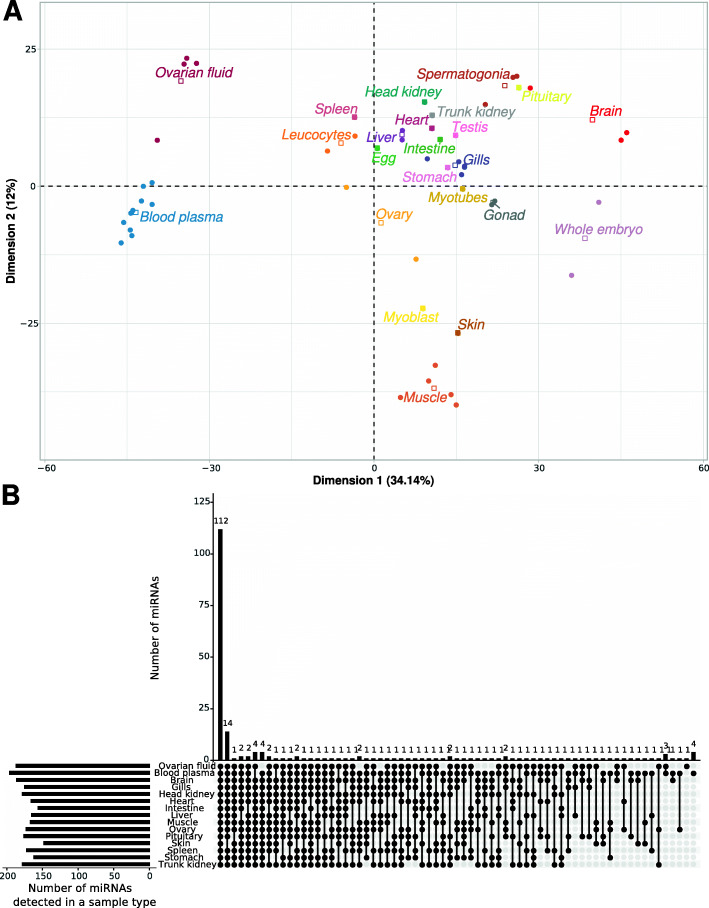
Detection of blood plasma and ovarian fluid miRNAs in various organs and cell types. **A** PCA analysis of normalized RPM counts supplied by Prost! using all samples (***N***=52). PCA was centered but not scaled. Corresponding data are available in additional file 4. **B** The number of circulating miRNAs detected in all possible combinations of analyzed libraries is displayed. Libraries in which miRNAs were detected are indicated by a black dot. miRNAs were considered present in an organ or a fluid if their normalized abundance exceeded 10 RPM, on average, for each type of sample. The analysis was limited to the two biological fluids (blood plasma and ovarian fluid) and a subset of 13 different female organs displayed in Fig. 3

When miRNA expression in fluids was analyzed together with expression data in the panel of 13 female organs from which they could originate, we observed that both blood plasma and ovarian fluid exhibited specific expression patterns as distinct as in the other analyzed organs, if not more. The heatmap presented in Fig. 3B clearly shows that, while sharing common strongly expressed miRNAs, each fluid was nonetheless characterized by the overabundance of several miRNAs (the yellow ones towards the top right of the panel) that were not overabundant in any organs analyzed in the present study (Additional file 1). The PCA analysis carried out using all available samples clearly illustrated that biological fluid miRNAomes, while distinct, were also clearly different from all other “solid” tissue and organ miRNAomes (Fig. 4A). When analyzing the presence of miRNAs in the different libraries, we observed that most miRNAs present in the blood plasma and ovarian fluid were also detected in most organs (Fig. 4B).

**Fig. 5:**
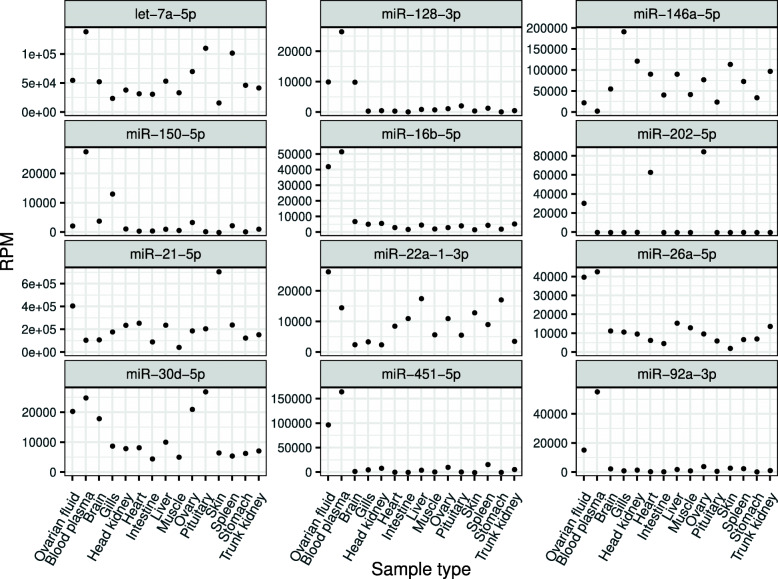
Normalized expression values of the most abundant c-miRNAs across organs and biological fluids. The analysis was performed using the two biological fluids (blood plasma and ovarian fluid) and a subset of 13 different female organs displayed in Fig. 3. Normalized RPM counts were averaged for all samples of a given sample type. miR-451-5p, let-7a-5p, miR-21-5p, miR-16b-5p, miR-30d-5p, and miR-26a-5p were among the 10 most abundant miRNAs for both blood plasma and ovarian fluid. miR-92a-3p, miR-150-5p, and miR-128-3p were among the most abundant miRNAs in the blood plasma. miR-202-5p, miR-22a-1-3p, and miR-146a-5p were among the most abundant miRNAs in ovarian fluid. Corresponding data are available in Additional file 1

When analyzing the expression of the miRNAs exhibiting strong expression in the ovarian fluid and blood plasma, we observed that among the 10 most abundant miRNAs in the ovarian fluid and plasma, seven (miR-451-5p, let-7a-5p, miR-21-5p, miR-16b-5p, miR-26a-5p, let-7e-5p, miR-30d-5p) were common to both fluids. In the blood plasma, miR-92a-3p, miR-150-5p, and miR-128-3p were the three other most abundant miRNAs. In the ovarian fluid, miR-202-5p, miR-22a-1-3p, and miR-146a-5p were the three other most abundant miRNAs. For miR-451-5p, miR-16b-5p, miR-26a-5p, and miR-92a-3p, a clear over abundance was observed in both fluids in comparison to organs (Fig. 5). In contrast, the other most abundant miRNAs in fluids were also highly abundant in at least one other organ (Fig. 5).

Together, these data indicate that c-miRNA repertoires in rainbow trout are complex. Similar to what was observed for miRNAs in different organs, c-miRNAomes in the blood plasma and ovarian fluid each exhibited specific expression profiles, with fluid-specific combinations of highly expressed miRNAs, major differences in miRNA abundances, and fluid-type-specific miRNAs in comparison to the other fluid and organs analyzed. These data suggest that the complexity of c-miRNAomes in the blood plasma and ovarian fluid results, at least in part, from the accumulation of miRNAs originating from a wide diversity of organs. The presence in body fluids of miRNAs that cannot be detected in other organs, or detected at much lower levels, suggests that these miRNAs originated from other sources that were not investigated here. These specific miRNAs could also have originated from miRNA-expressing cells present in these biological fluids or in their vicinity. For example, miR-451-5p could have originated from erythrocytes that greatly express this miRNA during the late stage of red-blood cell maturation [38–41] and miR-21-5p could have been expressed by the endothelial cells forming the vasculature [42]. Finally, it is also possible that the level of these miRNAs resulted from their progressive accumulation in these fluids over time permitted by their high stability in nuclease-rich fluids [43].

### Differences and similarities of ovarian fluid and blood plasma miRNA repertoires

**Fig. 6:**
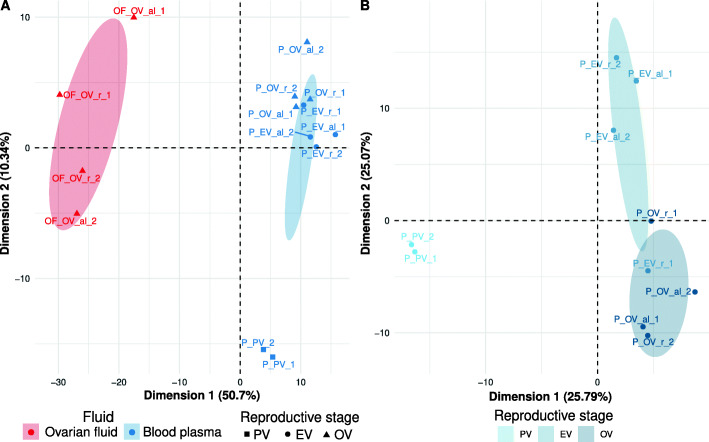
Principal Component Analyses of biological fluid c-miRNAs. PCAs were centered but not scaled and computed from normalized miRNAs counts (RPM) supplied by Prost!. Ellipses represent 95% confidence intervals and were drawn for conditions represented by at least three individuals. Points are samples named under the following pattern: “Fluid_Reproductive stage_feeding_replicate.” The value for “Fluid” is either the blood plasma (P) or the ovarian fluid (OF). The following reproductive stages were analyzed: pre-vitellogenesis (PV), early vitellogenesis (EV), and ovulation (OV). The feeding level was either ad libitum (al) or restricted (r). Corresponding data are available in Additional file 4. **A** PCA of all 10 blood plasma and 4 ovarian fluid samples. **B** PCA using only the blood plasma samples (*n*=10) collected at the three different reproductive stages

As indicated above, the blood plasma and ovarian fluid had many miRNAs in common. Marked differences between blood plasma and ovarian fluid miRNAomes, however, existed in terms of both miRNA profiles and expression of fluid-specific miRNAs. The PCA analysis of fluid samples only (Fig. 6A) clearly showed that the overall c-miRNA profiles differed between the blood plasma and ovarian fluid samples. Accordingly, 138 miRNAs were significantly differentially abundant between the ovarian fluid and blood plasma (Additional file 2). Among these 138 miRNAs, 67 were over-abundant in ovarian fluid and 71 in the blood plasma.

Our results are consistent with previous studies in humans showing distinct miRNA compositions in different body fluid types [4]. While the authors suggested a common origin for miRNAs present in the different body fluids, they also reported fluid-specific enrichment of several miRNAs, including in the blood plasma. In the present study, two miRNAs (miR-202-5p and miR-194b-5p) exhibited a dramatic, over 100-fold, enrichment in ovarian fluid in comparison to the blood plasma (Additional file 2). In contrast, two miRNAs (miR-460-5p and miR-365-2-5p) exhibited the opposite pattern. While the function of extracellular miRNAs remains unclear [43], it has been hypothesized that fluid-specific miRNAs could have regulatory functions in surrounding tissues [4]. In rainbow trout, as in many vertebrates, miR-202-5p is predominantly expressed in gonads [28, 44–48]. The strong abundance of miR-202-5p in ovarian fluid therefore agrees with its strong ovarian expression because the ovarian fluid, in which ovulated oocytes (i.e., unfertilized eggs) are held in the body cavity until spawning, is, at least in part, from ovarian origin [49]. Conversely, the blood perfuses all organs and transports molecules throughout the body, including in the ovaries [4] and many ovarian fluid components such as proteins are also known to be brought in from the blood [50, 51]. Ovarian fluid c-miRNAs may thus also, in part, be brought in the ovarian fluid via the blood, which would be consistent with the presence of a high proportion of common miRNAs in both fluids. In medaka, miR-202-5p plays a major role in female reproduction, specifically in the control of egg production and egg ability to be fertilized [44]. The overabundance of miR-202-5p in ovarian fluid, compared to the blood plasma, would be consistent with a physiological role of miR-202-5p in the ovarian fluid before, at, or after ovulation, a period associated with major events, including the final maturation of oocytes and the onset of the next reproductive cycle.

### Varying c-miRNA abundance in response to reproductive and metabolic states

**Fig. 7:**
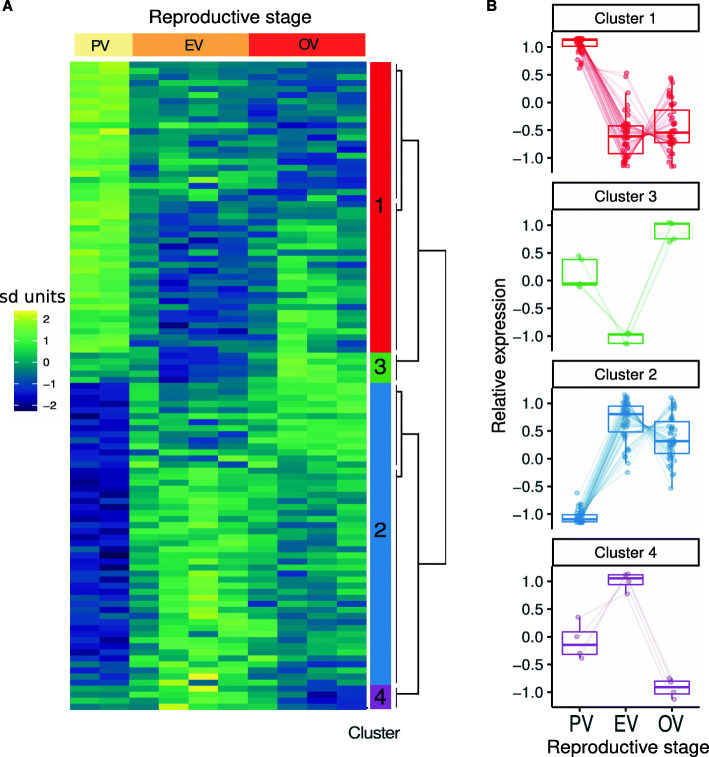
Expression profiling of c-miRNAs differentially expressed during the reproductive cycle. **A** Expression heatmap for c-miRNAs differentially expressed across reproductive stages. Expression values were log-transformed read counts of the 107 c-miRNAs differentially expressed during the reproductive cycle. The heatmap was scaled by row. Expression values were standardized and are expressed in standard deviation (sd) units. Expression dynamic clusters are indicated by colors in the rightmost column. The following reproductive stages were analyzed: pre-vitellogenesis (PV), early vitellogenesis (EV), and ovulation (OV). Corresponding data are available in Additional file 4. **B** Expression dynamics by cluster of the 107 differentially expressed c-miRNAs. All differentially expressed miRNAs were assigned to one of four clusters based on their expression dynamic during the reproductive period

In the present study, we also aimed at identifying blood plasma c-miRNAs that change in expression level during the reproductive cycle or in response to different metabolic states resulting from different feeding levels. The PCA analysis (Fig. 6B) revealed clear differences in blood plasma c-miRNAomes during the reproductive cycle. Differences were especially noticeable between samples taken at the beginning of the reproductive cycle (i.e., at previtellogenic stage) compared to samples taken later during the reproductive cycle. The statistical analysis led to the identification of 107 differentially abundant miRNAs during the reproductive cycle (Additional file 3). The heatmap of these miRNAs presented in Fig. 7A revealed four different clusters of miRNA expression profiles during the reproductive cycle. Most changes in expression occurred between previtellogenesis (PV) and early-vitellogenesis (EV). The differential expression analysis resulted in the identification of 48 downregulated (cluster 1) and 50 upregulated (cluster 2) miRNAs in PV compared to EV (Fig. 7B). In addition, we identified five miRNAs upregulated at ovulation (OV, cluster 3) and four miRNAs exhibiting the opposite pattern (cluster 4). Together, these results indicate that major changes occurred in the blood plasma c-miRNAome during the reproductive cycle and that a significant proportion of the blood plasma c-miRNAome (107 c-miRNAs, 55% of the overall blood plasma c-miRNAome) exhibited a differential abundance between at least two stages of the reproductive cycle. These results show that the blood plasma miRNAome exhibits marked stage-specific signatures during the reproductive cycle.

In contrast, we were not able to detect any significant differences in c-miRNA abundances in the blood plasma in response to metabolic levels (i.e., feeding level) (“al” for ad libitum and “r” for restricted diet in Fig. 7). This result could, however, originate from the low number of replicates (two sample pools per diet) that composed our RNA-seq dataset.

### Circulating blood plasma miRNAs as non-invasive biomarkers of metabolic and reproductive states

**Fig. 8:**
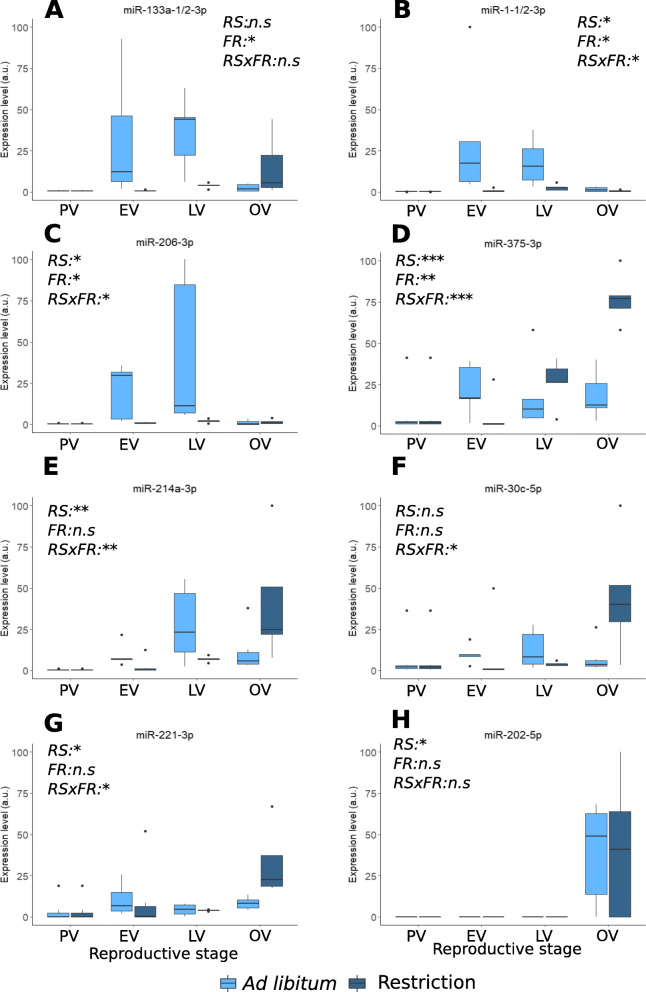
Blood plasma abundance of candidate biomarker c-miRNAs. Quantitative PCR analysis of selected miRNAs during the reproductive cycle and in response to feeding rate. The following reproductive stages were analyzed: pre-vitellogenesis (PV), early vitellogenesis (EV), late vitellogenesis (LV), and ovulation (OV). The feeding rate was either ad libitum (al) or restricted (r). Significant differences (ANOVA) in expression levels (arbitrary units, a.u.) are indicated for reproductive stage (RS), feeding rate (FR), and feeding rate—stage interactions (RS X FR). *p<0.05, **p<0.05, n.s. not significant (*p*>0.05). Replicates (*N*= 5 or 6) correspond to different individual fish. Corresponding data are available in Additional file 6

**Fig. 9:**
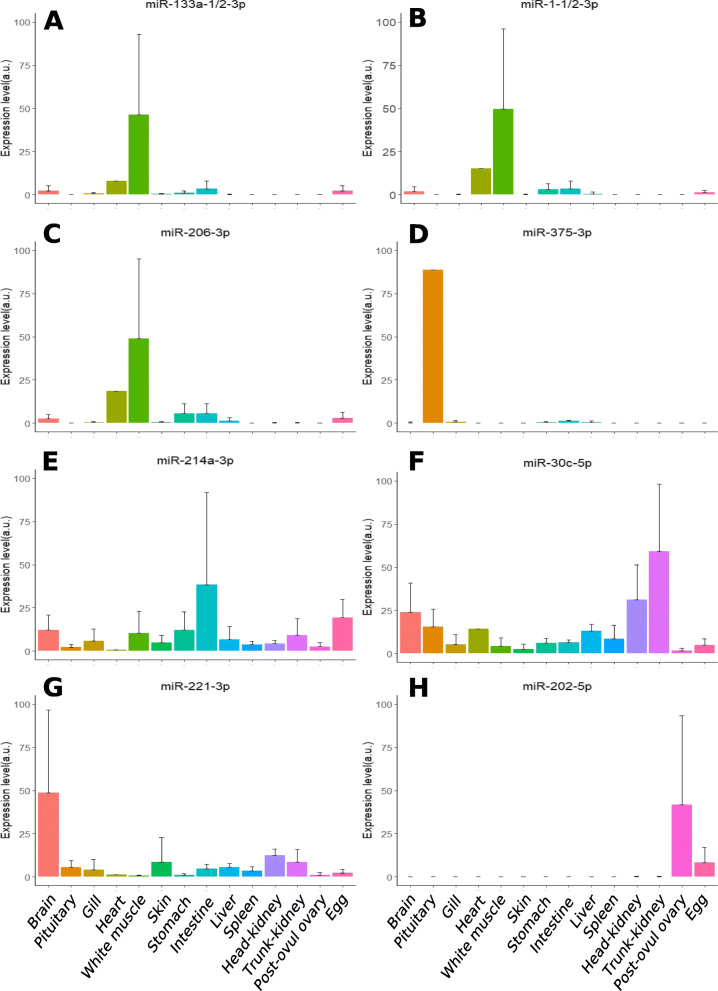
Candidate biomarker c-miRNA abundance in various organs. Quantitative PCR analysis of selected miRNAs in different organs. Replicates (*N*= 3, except for heart *N*=1) correspond to different individual fish. Means and standard error mean are displayed. Corresponding data are available in Additional file 6

To further evaluate the potential of blood plasma c-miRNAs to respond to differences in metabolic and reproductive states, we selected the most promising miRNAs (i.e., exhibiting the highest fold-changes between different metabolic levels or reproductive stages in our small RNA-seq data) and conducted an extended analysis of their expression by quantitative PCR (QPCR) using five individual replicates and an additional time point during the reproductive cycle (Late vitellogenesis, LV) (Fig. 8). The potential origin of these candidate c-miRNAs was also analyzed by QPCR in a panel of organs to shed light on their possible origin of expression (Fig. 9). Quantitative PCR demonstrated that selected biomarker c-miRNA candidates exhibited highly significant changes in their blood plasma abundance throughout the reproductive cycle. In most cases, these changes occurred in a feeding level-dependent manner, indicating that circulating miRNA levels in the blood plasma can be deeply influenced by metabolism. Among analyzed candidate biomarkers, miR-1-1/2-3p, miR-133a-1/2-3p, and miR-206-3p exhibited a similar pattern throughout the reproductive cycle with a dramatic increase in blood plasma abundance during vitellogenesis (i.e., the reproductive phase characterized by major yolk protein uptake from the blood stream by the oocyte) when fish were fed ad libitum but not when the food was restricted (Fig. 8A–C). When investigating the organs expressing these three miRNAs, we observed a predominant expression in skeletal muscle (Fig. 9A–C). These miRNAs are known to be muscle-specific miRNAs, often referred to as “myomiRs” [52], and have been associated with myogenesis and with various biological processes in the skeletal muscle and heart [53–62]. In Nile tilapia, an increase in the expression of these three myomiRs was observed in muscles throughout the fish life [63]. The association between these miRNAs and active myogenesis thus appears to be evolutionarily conserved in vertebrates. A higher level of blood plasma myomiRs in well-fed animals compared to animals under a restricted diet would be consistent with the significant increase in growth rate observed in the present individuals when fed ad libitum [27]. Together, these observations suggest that blood plasma levels of miR-1-1/2-3p, miR-133a-1/2-3p, and miR-206-3p have the potential to identify episodes of active myogenesis. Under the hypothesis that these potential biomarker myomiRs reflect muscle growth rate, which requires experimental validation using additional samples and individuals held in a variety of experimental conditions, this result could offer a wide range of possible applications. For wild population management, these biomarker candidates could for instance offer the possibility to assess the quality of an ecosystem through the ability to monitor fish growth throughout the year. In aquaculture, it could allow fine phenotyping of muscle growth in response to specific diets or rearing conditions. More importantly, these biomarker candidates could allow to specifically question muscle growth in comparison to global body growth that can be influenced by the development of other tissues such as fat deposits, an information that is currently not easily accessible without sacrificing the fish.

Among the miRNAs that we investigated by QPCR to assess their potential use in non-invasive phenotyping, four c-miRNAs exhibited significant changes in their blood plasma abundance in response to feeding rate, either globally in the case of miR-375-3p (Fig. 8D) or in interaction with the reproductive stage in the case of miR-214a-3p, miR-30c-3p, and miR-221-3p (Fig. 8E–G). The latter, miR-214-a-3p, miR-30c-3p, and miR-221-3p, was also expressed in a wide variety of organs (Fig. 9E–G), making it hazardous to speculate on their organ of origin and the biological processes in which they may be involved. The expression profiles of these c-miRNAs, however, indicate that they could be used, most likely in combination with other c-miRNAs, to estimate the metabolic state of the fish at a given reproductive stage. Interestingly, these three c-miRNAs have been used as blood plasma biomarkers for several human pathologies such as cancer (liver, prostate, ovarian, and pancreatic cancers) and cardiovascular and renal diseases [64–69]. In contrast, the highly predominant expression of miR-375-3p in the pituitary (Fig. 9D), which is consistent with existing data in other vertebrate species [70, 71] can tentatively be associated with the neuroendrocrine control of biological processes such as nutrition and reproduction. This c-miRNA exhibited highly significant differences in blood plasma abundance in response to feeding rate both globally and in a reproductive-stage-dependent manner (Fig. 8). The difference in miR-375-3p levels in response to feeding rate was especially marked during late vitellogenesis and ovulation period. In other animal species, miR-375-3p has been implicated in the regulation of insulin [71] but a role in reproduction metabolism has also been suggested [70, 72]. Even though the role of miR-375-3p in animal reproduction remains unclear, this miRNA appeared to be abundant in the blood plasma and highly responsive to changes in reproductive and metabolic states in female rainbow trout. For these reasons, miR-375-3p is a highly promising candidate biomarker for non-invasive phenotyping of neuroendocrine response in rainbow trout and possibly other animal species.

Among the c-miRNAs that we monitored by QPCR throughout the reproductive cycle, miR-202-5p had the most striking profile (Fig. 8H). Independent of the feeding regime, miR-202-5p exhibited a dramatic increase in its blood plasma abundance at ovulation and was also among the most highly abundant c-miRNAs in ovarian fluid according to the small RNA-seq data. In fish, miR-202-5p plays a major role in reproduction and female medaka lacking expression of miR-202-5p produced fewer eggs and of lesser quality [44]. It is therefore possible that miR-202-5p in the blood plasma and in ovarian fluid plays an important biological role around the time of ovulation that would require further investigations. As already described in rainbow trout, teleost fishes and other vertebrates, miR-202-5p is predominantly expressed in the ovary and was also detected in unfertilized eggs (Fig. 9H) [28, 44, 45, 73, 74]. In the rainbow trout ovary, miR-202-5p was differentially expressed during oogenesis with a peak of expression during vitellogenesis followed by a progressive decrease during final oocyte maturation [75]. The profile of miR-202-5p in the blood plasma reported here with a peak of expression at ovulation thus differs from its ovarian expression. It is possible that this discrepancy results from the delay between expression in the ovary during vitellogenesis and accumulation in the blood plasma during periovulatory period. This is, however, unlikely given the 2–3-month periods between mid-vitellogenesis and ovulation. The sharp increase in blood plasma miR-202-5p levels at ovulation (Fig. 8D), in contrast, suggests a release during the periovulatory period, either from the ovary or from the eggs. It is thus possible that a dynamic accumulation of miR-202-5p in the blood plasma occurs either immediately prior to or following ovulation. Under this hypothesis, circulating miR-202-5p levels could serve as a biomarker to predict approaching ovulation, if the accumulation in the blood plasma occurs prior to ovulation, or to estimate post-ovulatory egg ageing, if the accumulation in the blood plasma occurs at or after ovulation. In both cases, this c-miRNA would be of major interest as a non-invasive phenotyping biomarker enabling, in aquaculture or wild resource management settings, the selection of females that are close to or at ovulation to prevent the occurrence of post-ovulatory ageing of the eggs, a phenomenon associated with a dramatic decrease in egg quality [76].

Together, both small RNA-seq and QPCR data revealed that the levels of selected circulating miRNAs exhibited major differences during the female rainbow trout reproductive cycle and, for some of them, also in response to changes in metabolic state. Some of these c-miRNAs therefore appear to be highly relevant candidate biomarkers that could serve for non-invasive phenotyping of sexual maturation (i.e., progress into the reproductive cycle) and episodes of muscle growth. These results are consistent with recent observations in rainbow trout showing that specific c-miRNAs were differentially abundant in the blood plasma, mucus, and surrounding water very rapidly after a stressful event [25]. Together, these observations highlight the strong potential of c-miRNAs to serve as biomarkers and non-invasive indicators of stress, reproductive and metabolic states, and myogenic activity. Further investigations are however needed to explore their potential in other physiological and pathological contexts in various fish species and to validate them as biomarkers. The identification of other biomarker c-miRNAs, such as markers of viral and bacterial infections, would allow the collection of a panel of relevant complementary information from a single blood sample and thus offer tremendous phenotyping possibilities.

## Conclusion

In the present study, we provide a reference rainbow trout miRNA repertoire annotation that was previously incomplete in this species and the first comprehensive analysis of blood plasma and ovarian fluid miRNAomes in a fish species. We show that biological fluid miRNAomes are extremely diverse and encompass a high proportion of the overall miRNAome of the species. While sharing common miRNAs, blood plasma and ovarian fluid miRNAomes nevertheless exhibited marked differences with fluid-specific combinations of highly abundant miRNAs and a few fluid-specific miRNAs. In addition, our data suggest that the complexity of c-miRNAomes in blood plasma and ovarian fluid originates, at least in part, from the accumulation of miRNAs expressed in a wide diversity of organs. Our results also raise the question of fluid-specific miRNAs that could result from a fluid-dependent accumulation of some c-miRNAs over time. We further showed that the blood plasma exhibited major changes in c-miRNA abundances depending on the metabolic and the reproductive state. We subsequently identified a subset of three evolutionarily conserved myomiRs (miR-1-1/2-3p, miR-133a-1/2-3p, and miR-206-3p) that accumulated in the blood plasma in response to high feeding levels and thus appear as strong candidate biomarkers of active myogenesis. We also identified miR-202-5p as a candidate biomarker of ovulation that could be used to predict ovulation and thus the preservation of egg quality. Despite a lack of clear understanding of the biological roles of c-miRNAs, these highly promising results highlight the potential of c-miRNAs as physiologically relevant biomarkers and pave the way for the use of c-miRNAs for non-invasive phenotyping in many fish species.

## Methods

### Experimental design and fluid sampling

To generate contrasting physiological conditions, two feeding strategies were used throughout the reproductive cycle in rainbow trout (Fig. 1). Females were either fed ad libitum or at 80% of ad libitum (restriction). These two feeding regimes were used to trigger contrasted metabolic states that induced significantly higher increases in fish weight and condition factor in fish fed ad libitum compared to fish under the restricted diet as previously described [27]. The blood plasma samples were obtained at four stages of the reproductive cycle: previtellogenesis (PV), early-vitellogenesis (EV), late-vitellogenesis (LV), and ovulation (OV). The ovarian fluid was sampled at ovulation. Reproductive stages were estimated based on existing data on rainbow trout reproductive cycle [77]. Ovulation was checked once a week and fish were sampled 2 days after ovulation was detected. Ovulation (OV) stage thus corresponds to fish for which ovulated eggs have been present in the body cavity for 2 to 9 days. The blood samples were collected from the caudal vein using EDTA-coated syringes (sodium EDTA, 10%). The blood samples were centrifuged (3000 g, 15 min, 4°C), and the plasma samples were aliquoted, frozen in liquid nitrogen, and stored at −80°C until analysis. Ovarian fluid was collected after manual stripping of the eggs from ovulated femalesover a mesh screen. The collected ovarian fluid was then centrifuged (3000 g, 15 min, 4°C) to pellet cells and debris, aliquoted, frozen in liquid nitrogen, and stored at −80°C until analysis.

### RNA preparation

For small-RNA sequencing (sRNA-seq), RNA extraction was carried out using pooled samples of the blood plasma and ovarian fluid. For both fluids, each pool was made with equal volumes from 50 individual samples. A total of 14 pooled samples were used for small RNA sequencing (Fig. 1). For the blood plasma, five experimental conditions were analyzed: a first stage at the beginning of the experiment common to both feeding rates, and two stages during oogenesis for each of the two feeding levels conditions (ad libitum vs restriction). For each condition, two pools originating from 50 different individuals were analyzed resulting in a total of 10 small RNA sequencing libraries (Fig. 1). For ovarian fluid, each feeding level was sampled in duplicates resulting in a total of four small RNA sequencing libraries (Fig. 1).

For quantitative PCR (QPCR) validation of differential expression, extractions were carried out on individual blood plasma samples. RNA was extracted from five samples per condition and a total of 7 conditions were analyzed (Fig. 1). The validation by QPCR was carried out only on the blood plasma samples. Samples were randomly taken from all four time-points during oogenesis and for each of the two feeding conditions (ad libitum vs restriction).

For both small RNA-seq and QPCR, fluid samples were homogenized in Trizol reagent (Macherey-Nagel, Düren, Germany) at a ratio of 400 μL of fluid per milliliter of reagent and total RNA was extracted according to manufacturer’s instructions. During the RNA extraction protocol, glycogen was added to each sample to facilitate visualization of precipitated RNAs. Expression of selected candidate miRNAs was analyzed by QPCR in 14 different organs and tissues (brain, pituitary, gills, heart, white muscle, skin, stomach, intestine, liver, spleen, head kidney, trunk kidney, post-ovulatory ovary, and egg) that were sampled from three ovulated females. Tissues were homogenized in Trizol reagent at a ratio of 100 mg per milliliter of reagents, and total RNA was extracted according to the manufacturer's instructions.

### Small RNA sequencing

Illumina sequencing libraries were constructed using the NEXTflex small RNA kit v3 (Bioo Scientific). Starting from 1 μg of total RNA, an adapter was ligated on the 3′ end of the small RNAs. A second adapter was ligated to the 5′ end. Ligated small RNAs were subjected to reverse transcription using M-MuLV transcriptase and a RT primer complementary to the 3′ adapter. PCR amplification (16 cycles) was performed on the cDNA using a universal primer and a barcoded primer. Final size selection was performed on 3% gel cassette on a Pippin HT between 126pb and 169pb. Sequencing (single read 50 nucleotides) was performed using a HiSeq2500 (Illumina) with SBS (Sequence By Synthesis) technique. After quality filter, a total of over 187 million reads were obtained with a number of read per library ranging from 12.1 to 15.0 millions. Raw reads were deposited into NCBI Sequence Read Archive under accession number PRJNA631932. Reads were trimmed of the adaptor sequence GCCTTGGCACCCGAGAATTCCA and of the random primers using Cutadapt [78].

### Establishment of a reference rainbow trout miRNAome

The rainbow trout miRNAome annotation was established using *Prost!* [32], which was run on the rainbow trout reference genome (NCBI RefSeq assembly accession GCF_002163495.1) and with all ovarian fluid and blood plasma reads generated in the present study. The annotation was performed in comparison with a set of five manually curated fish mature miRNA and miRNA hairpin sequences (*Lepisosteus oculatus, Danio rerio*, *Poecilia mexicana*, *Gasterosteus aculeatus*, and *Chaenocephalus aceratus*) [31, 33, 34]. *Prost!* was run using the zebrafish as focal species and default settings (minimum read count of 20, sequence size comprised between 17 and 25). The resulting set of annotated genomic locations was then curated using the recommendations of *Prost!* documentation. First, reads with at least one mismatched nucleotide to the genome, or aligning to more than 20 locations, were not considered for annotation. Then, homology to previously described miRNAomes was used to build a set of annotated mature miRNA sequences in rainbow trout. When miRNAs displayed sequence variations aligning to the same locus or loci (i.e., isomiRs) [79], the most abundant isomiR for the locus was the one annotated.

To provide a comprehensive rainbow trout miRNAome annotation, this set was further extended by rerunning *Prost!* with the same settings but on samples originating from a wide variety of tissues, organs, and cell types [28] and using a combination of the above described rainbow trout preliminary annotation and all existing teleost miRNA mature and hairpin sequences in miRBase. The resulting rainbow trout miRNAome annotation is freely available here: https://github.com/INRAE-LPGP/microRNA.

### miRNA quantification and differential expression analyses

*Prost!* was run using the newly obtained rainbow trout miRNA annotation, all the fluid sequencing samples, and identical settings as for the annotation steps. Normalized miRNA counts in reads per million (RPM) were extracted from the “compressed by annotation” *Prost!* output tab. When *Prost!* identified multiple potential annotations for a given isomiR, counts for this sequence were distributed evenly between the possible annotations. Normalized counts in libraries from the same sample type were averaged, and miRNAs for which normalized abundance was greater than 10 RPM were considered expressed in a given tissue. Raw and normalized read counts are provided in Additional file 4.

To evaluate putative organ origin of circulating miRNAs, we associated each c-miRNA to the organ in which it was the more abundant, under the hypothesis that it represents the organ from which it was the most likely to originate, or at least one the major contributors. For this analysis, we excluded several types of samples, including spermatogonia, testis, eggs, whole embryo, myoblasts, myotubes, and leucocytes. Each c-miRNA was therefore assigned to one of 13 organs (brain, gills, heart, head kidney, intestine, liver, muscle, ovary, pituitary, skin, spleen, stomach, trunk kidney).

Expression data across organs were visualized using a heatmap based on the expression matrix centered and reduced by row and generated with the R package *heatmaply* [80]. Expression within the 14 fluid samples was visualized by performing a Principal Component Analyses (PCA) on log-transformed DESeq2 counts. To account for the greater importance of abundant miRNAs, the PCAs were centered but not scaled. PCAs were computed using the R package *FactoMineR* [81] and 95% confidence ellipses associated to the different condition were drawn using the plotellipses function. In the case of previtellogenesis blood plasma samples, only two samples were available; therefore, no ellipse could be drawn.

Differential expression analyses were performed using DESeq2 [82] and raw counts from all 14 fluid miRNA expression data. For each differential expression test, log fold changes were corrected using lfcShrink(type=”apeglm”), and *p* values were adjusted using the FDR method to account for multiple testing.

To identify miRNAs differentially expressed between the blood plasma and ovarian fluid samples, a model was built using all samples and considering all possible effects (~Sample + Feeding + Time). Differential expression was tested using a blood plasma vs*.* ovarian fluid contrast (i.e., “Sample” contrast). Differential expression of miRNAs between ad libitum and restricted feeding was further tested independently in the blood plasma and ovarian fluid samples with the same parameters.

Differential expression across the three considered reproductive stages was evaluated only for the 10 blood plasma samples because the ovarian fluid was sampled only at ovulation. Differential expression was tested using a likelihood ratio test between the “~ Time + Feeding” model and the “~Feeding” model. Afterwards, expression trajectories for the differentially expressed miRNAs were clustered using the function degPatterns of the R package *DEGReport* [83] on a regularized log-transformed DESeq2 count matrix.

### QPCR validation of miRNA expression and statistical analysis

Expression of selected miRNAs in fluids and organs was assessed using the TaqMan Advanced miRNA Assay (Applied Biosystems, ThermoFisher) according to the manufacturer’s instructions and using custom-made rainbow trout probes. Synthetic cel-miR-39 mimic (miScript miRNA Mimics, QIAGEN) was spiked in each RNA sample at a ratio of 11 fmol per μg of total RNA and used as an exogenous control for normalization. Briefly, 1.75 ng of total RNA was used for the initial poly(A) tailing, ligation of [something], and reverse transcription reactions to synthesize the cDNAs of all miRNAs followed by a pre-amplification step. The assays were carried out in a reaction mix of 10 μL containing 2.5 μL of cDNA (diluted 5 times), 5 μL of 2X Fast Advanced Master mix (Applied Biosystems, USA), 0.5 μL of TaqMan Advanced miRNA Assay (20X) (Applied Biosystems, USA), and 2 μL of DNAse/RNAse-free water. Quantitative RT-PCR was performed using the LightCycler 480 System (Roche Life Science) with the following conditions: 95°C for 20 s, and 40 cycles of 95°C for 1 s and 60°C for 20 s. The relative expression of miRNA within a sample set was calculated from a standard curve using LightCycler 480 System software release 1.5.1.62. All RT-QPCR reactions were performed in duplicates. Data were normalized by the cel-miR-39 detection levels. Sequences of the miRNA probes are provided in Additional file 5. Expression levels measured by RT-QPCR in Figs. 7 and 8 were given as means with standard deviations across biological replicates. Statistical analyses of the data were carried out using R studio software. Two-way ANOVA was performed to assess the effect of feeding level and reproductive stage on miRNA abundances.

## Supplementary Information

**Additional file 1.** Averaged normalized read counts (RPM) for all annotated miRNAs detected in biological fluids and organs.

**Additional file 2.** Differentially expressed miRNAs between ovarian fluid and blood plasma samples.

**Additional file 3.** Differentially expressed miRNAs in blood plasma during reproductive cycle or in response to feeding rate.

**Additional file 4.** Normalized and raw read counts in all analyzed libraries.

**Additional file 5.** QPCR primer sequences.

**Additional file 6.** Individual data used in Fig. 8 and 9.

## Data Availability

All data generated or analyzed during this study are included in this published article, its supplementary information files, and publicly available repositories. The datasets generated and analyzed during the present study are available in NCBI SRA repository https://www.ncbi.nlm.nih.gov/sra/PRJNA631932 (rainbow trout biological fluids) and https://www.ncbi.nlm.nih.gov/bioproject/PRJNA227065 (rainbow trout tissues, organs, cell types, and embryos). The rainbow trout mature miRNAs annotated in this study are available here: https://github.com/INRAE-LPGP/microRNA.

## References

[1] Ambros V (2004). The functions of animal microRNAs. Nature..

[2] Alvarez-garcia I, Miska EA (2005). MicroRNA functions in animal development and human disease. Development..

[3] Allegra A, Alonci A, Campo S, Penna G, Petrungaro A, Gerace D, Musolino C (2012). Circulating microRNAs: new biomarkers in diagnosis, prognosis and treatment of cancer (Review). Int J Oncol.

[4] Weber J, Baxter D, Zhang S, Huang DY, Huang KH, Lee M, Galas DJ, Wang K (2010). The microRNA spectrum in 12 body fluids. Mol Diagnostics Genet.

[5] Etheridge A, Lee I, Hood L, Galas D, Wang K (2011). Extracellular microRNA : a new source of biomarkers. Mutat Res Fundam Mol Mech Mutagen.

[6] Hanke M, Hoefig K, Merz H, Feller A, Kausch I, Jocham D, Warnecke JM, Sczakiel G (2010). A robust methodology to study urine microRNA as tumor marker : microRNA-126 and microRNA-182 are related to urinary bladder cancer. Urol Oncol.

[7] Park NJ, Zhou H, Elashoff D, Henson BS, Kastratovic DA, Abemayor E, Wong DT (2009). salivary microRNA : discovery, characterization, and clinical utility for oral cancer detection. Clin Cancer Res.

[8] Mitchell PS, Parkin RK, Kroh EM, Fritz BR, Wyman SK, Pogosova-Agadjanyan EL, Peterson A, Noteboom J, O’Briant KC, Allen A, Lin DW, Urban N, Drescher CW, Knudsen BS, Stirewalt DL, Gentleman R, Vessella RL, Nelson PS, Martin DB, Tewari M (2008). Circulating microRNAs as stable blood-based markers for cancer detection. Proc Natl Acad Sci U S A.

[9] Larrea E, Sole C, Manterola L, Goicoechea I, Armesto M, Arestin M, et al. New concepts in cancer biomarkers: circulating miRNAs in liquid biopsies. Int J Mol Sci. 2016;17. 10.3390/ijms17050627. 10.3390/ijms17050627 PMC4881453 27128908

[10] Cheng G, Luo R, Hu C, Cao J, Jin Y (2013). Deep sequencing-based identification of pathogen-specific microRNAs in the plasma of rabbits infected with Schistosoma japonicum. Parasitology..

[11] Hansen EP, Kringel H, Thamsborg SM, Jex A, Nejsum P (2016). Profiling circulating miRNAs in serum from pigs infected with the porcine whipworm, Trichuris suis. Vet Parasitol.

[12] Taxis TM, Bauermann FV, Ridpath JF, Casas E (2017). Circulating microRNAs in serum from cattle challenged with bovine viral diarrhea virus. Front Genet.

[13] Spornraft M, Kirchner B, Michael WP, Riedmaier I (2015). The potential of circulating extracellular small RNAs (smexRNA) in veterinary diagnostics-Identifying biomarker signatures by multivariate data analysis. Biomol Detect Quantif.

[14] Han W, Zhu Y, Su Y, Li G, Qu L, Zhang H, Wang K, Zou J, Liu H (2016). High-throughput sequencing reveals circulating miRNAs as potential biomarkers for measuring puberty onset in chicken (Gallus gallus). PLoS One.

[15] Ioannidis J, Donadeu FX (2016). Circulating microRNA profiles during the bovine oestrous cycle. PLoS One.

[16] Ioannidis J, Donadeu FX. Circulating miRNA signatures of early pregnancy in cattle. BMC Genomics. 2016:1–12. 10.1186/s12864-016-2529-1. 10.1186/s12864-016-2529-1 PMC4778341 26939708

[17] Pohler KG, Green JA, Moley LA, Gunewardena S, Hung WT, Payton RR, Hong X, Christenson LK, Geary TW, Smith MF (2017). Circulating microRNA as candidates for early embryonic viability in cattle. Mol Reprod Dev.

[18] Muroya S, Ogasawara H, Hojito M (2015). Grazing affects exosomal Circulating microRNAs in cattle. PLoS One.

[19] Muroya S, Shibata M, Hayashi M, Oe M, Ojima K (2016). Differences in circulating microRNAs between grazing and grain-fed wagyu cattle are associated with altered expression of intramuscular microRNA, the Potential Target PTEN, and Lipogenic Genes. PLoS One.

[20] Fernández I, Fernandes JM, Roberto V, Kopp M, Oliveira C, Riesco MF, Dias J, Cox CJ, Cancela ML, Cabrita E, Gavaia P (2019). Circulating small non-coding RNAs provide new insights into vitamin K nutrition and reproductive physiology in teleost fish. BBA Gen Subj.

[21] Ahanda MLE, Zerjal T, Dhorne-Pollet S, Rau A, Cooksey A, Giuffra E (2014). Impact of the genetic background on the composition of the chicken plasma MiRNome in response to a stress. PLoS One.

[22] Ma J, Li Y, Wu M, Zhang C, Che Y, Li W, Li X (2018). Serum immune responses in common carp (Cyprinus carpio L.) to paraquat exposure: the traditional parameters and circulating microRNAs. Fish Shellfish Immunol.

[23] Zheng Y, Chen KL, Zheng XM, Li HX, Wang GL (2014). Identification and bioinformatics analysis of microRNAs associated with stress and immune response in serum of heat-stressed and normal Holstein cows. Cell Stress Chaperones.

[24] Kong Z, Zhou C, Li B, Jiao J, Chen L, Ren A, Jie H, Tan Z (2019). Integrative plasma proteomic and microRNA analysis of Jersey cattle in response to high-altitude hypoxia. J Dairy Sci.

[25] Ikert H, Lynch MDJ, Doxey AC, Giesy JP, Servos MR, Katzenback BA, Craig PM (2021). High throughput sequencing of MicroRNA in rainbow trout plasma, mucus, and surrounding water following acute stress. Front Physiol.

[26] Bizuayehu TT, Babiak I. Heterogenic origin of micro RNAs in atlantic salmon (Salmo salar) seminal plasma. Int J Mol Sci. 2020;21. 10.3390/ijms21082723. 10.3390/ijms21082723 PMC7216159 32326572

[27] Cardona E, Bugeon J, Guivarc’h F, Goardon L, Panserat S, Labbé L, Corraze G, Skiba-Cassy S, Bobe J (2019). Positive impact of moderate food restriction on reproductive success of the rainbow trout Oncorhynchus mykiss. Aquaculture..

[28] Juanchich A, Bardou P, Rué O, Gabillard JC, Gaspin C, Bobe J, Guiguen Y (2016). Characterization of an extensive rainbow trout miRNA transcriptome by next generation sequencing. BMC Genomics.

[29] Desvignes T, Sydes J, Montfort J, Bobe J, Postlethwait JH (2021). Evolution after whole genome duplication: teleost microRNAs. Mol Biol Evol.

[30] Braasch I, Gehrke AR, Smith JJ, Kawasaki K, Manousaki T, Pasquier J, Amores A, Desvignes T, Batzel P, Catchen J, Berlin AM, Campbell MS, Barrell D, Martin KJ, Mulley JF, Ravi V, Lee AP, Nakamura T, Chalopin D, Fan S, Wcisel D, Caestro C, Sydes J, Beaudry FEG, Sun Y, Hertel J, Beam MJ, Fasold M, Ishiyama M, Johnson J, Kehr S, Lara M, Letaw JH, Litman GW, Litman RT, Mikami M, Ota T, Saha NR, Williams L, Stadler PF, Wang H, Taylor JS, Fontenot Q, Ferrara A, Searle SMJ, Aken B, Yandell M, Schneider I, Yoder JA, Volff JN, Meyer A, Amemiya CT, Venkatesh B, Holland PWH, Guiguen Y, Bobe J, Shubin NH, Di Palma F, Alföldi J, Lindblad-Toh K, Postlethwait JH (2016). The spotted gar genome illuminates vertebrate evolution and facilitates human-teleost comparisons. Nat Genet.

[31] Kim BM, Amores A, Kang S, Ahn DH, Kim JH, Kim IC, Lee JH, Lee SG, Lee H, Lee J, Kim HW, Desvignes T, Batzel P, Sydes J, Titus T, Wilson CA, Catchen JM, Warren WC, Schartl M, Detrich HW, Postlethwait JH, Park H (2019). Antarctic blackfin icefish genome reveals adaptations to extreme environments. Nat Ecol Evol.

[32] Desvignes T, Batzel P, Sydes J, Eames BF, Postlethwait JH (2019). miRNA analysis with Prost! reveals evolutionary conservation of organ-enriched expression and post-transcriptional modifications in three-spined stickleback and zebrafish. Sci Rep.

[33] Kelley JL, Desvignes T, McGowan KL, Perez M, Rodriguez LA, Brown AP, et al. microRNA expression variation as a potential molecular mechanism contributing to adaptation to hydrogen sulphide. J Evol Biol. 2020:jeb.13727. 10.1111/jeb.13727. 10.1111/jeb.13727 33124163

[34] Desvignes T, Beam MJ, Batzel P, Sydes J, Postlethwait JH (2014). Expanding the annotation of zebrafish microRNAs based on small RNA sequencing. Gene..

[35] Kozomara A, Birgaoanu M, Griffiths-Jones S (2019). MiRBase: from microRNA sequences to function. Nucleic Acids Res.

[36] Fromm B, Domanska D, Høye E, Ovchinnikov V, Kang W, Aparicio-Puerta E, Johansen M, Flatmark K, Mathelier A, Hovig E, Hackenberg M, Friedländer MR, Peterson KJ (2020). MirGeneDB 2.0: the metazoan microRNA complement. Nucleic Acids Res.

[37] Cadonic IG, Ikert H, Craig PM (2020). Comparative Biochemistry and Physiology - Part D Acute air exposure modulates the microRNA abundance in stress responsive tissues and circulating extracellular vesicles in rainbow trout ( Oncorhynchus mykiss ). Comp Biochem Physiol - Part D.

[38] Pase L, Layton JE, Kloosterman WP, Carradice D, Waterhouse PM, Lieschke GJ (2009). miR-451 regulates zebrafish erythroid maturation in vivo via its target gata2. Blood.

[39] Cifuentes D, Xue H, Taylor DW, Patnode H, Mishima Y, Cheloufi S, Ma E, Mane S, Hannon GJ, Lawson ND, Wolfe SA, Giraldez AJ (2010). A novel miRNA processing pathway independent of dicer requires argonaute2 catalytic activity. Science (80- ).

[40] Rasmussen KD, Simmini S, Abreu-Goodger C, Bartonicek N, Di Giacomo M, Bilbao-Cortes D, Horos R, Von Lindern M, Enright AJ, O’Carroll D (2010). The miR-144/451 locus is required for erythroid homeostasis. J Exp Med.

[41] Yang S, Maurin T, Robine N, Rasmussen KD, Jeffrey KL, Chandwani R, Papapetrou EP, Sadelain M, O’Carroll D, Lai EC (2010). Conserved vertebrate mir-451 provides a platform for Dicer-independent, Ago2-mediated microRNA biogenesis. Proc Natl Acad Sci U S A.

[42] Banjo T, Grajcarek J, Yoshino D, Osada H, Miyasaka KY, Kida YS, Ueki Y, Nagayama K, Kawakami K, Matsumoto T, Sato M, Ogura T (2013). Haemodynamically dependent valvulogenesis of zebrafish heart is mediated by flow-dependent expression of miR-21. Nat Commun.

[43] Turchinovich A, Weiz L, Burwinkel B (2012). Extracellular miRNAs : the mystery of their origin and function. Trends Biochem Sci.

[44] Gay S, Bugeon J, Bouchareb A, Henry L, Delahaye C, Legeai F, Montfort J, Le Cam A, Siegel A, Bobe J, Thermes V (2018). MiR-202 controls female fecundity by regulating medaka oogenesis. PLoS Genet.

[45] Zhang J, Liu W, Jin Y, Jia P, Jia K, Yi M (2017). MiR-202-5p is a novel germ plasm-specific microRNA in zebrafish. Sci Rep.

[46] Armisen J, Gilchrist MJ, Wilczynska A, Standart N, Miska EA (2009). Abundant and dynamically expressed miRNAs, piRNAs, and other small RNAs in the vertebrate Xenopus tropicalis. Genome Res.

[47] Landgraf P, Rusu M, Sheridan R, Sewer A, Iovino N, Aravin A, Pfeffer S, Rice A, Kamphorst AO, Landthaler M, Lin C, Socci ND, Hermida L, Fulci V, Chiaretti S, Foà R, Schliwka J, Fuchs U, Novosel A, Müller RU, Schermer B, Bissels U, Inman J, Phan Q, Chien M, Weir DB, Choksi R, De Vita G, Frezzetti D, Trompeter HI, Hornung V, Teng G, Hartmann G, Palkovits M, Di Lauro R, Wernet P, Macino G, Rogler CE, Nagle JW, Ju J, Papavasiliou FN, Benzing T, Lichter P, Tam W, Brownstein MJ, Bosio A, Borkhardt A, Russo JJ, Sander C, Zavolan M, Tuschl T (2007). A mammalian microRNA expression atlas based on small RNA library sequencing. Cell.

[48] Bannister SC, Smith CA, Roeszler KN, Doran TJ, Sinclair AH, Tizard MLV (2011). Manipulation of estrogen synthesis alters MIR202* expression in embryonic chicken gonads. Biol Reprod.

[49] Lahnsteiner F, Weismann T, Patzner RA. Composition of the ovarian fluid in 4 salmonid species : Oncorhynchus mykiss, Salmo trutta f lacustris, Saivelinus alpinus and Hucho hucho. Reprod Nutr Dev. 1995. 10.1051/rnd:19950501 8526977

[50] Matsubara T, Hara A, Takano K (1985). Immunochemical identification and purification of coelomic fluid-specific protein in chum salmon (Oncorhynchus keta). Comp Biochem Physiol.

[51] Matsubara T, Hara A, Takano K (1993). Immunohistochemical localization of coelomic fluid-specific protein in the coelomic epithelium and mesovarium of Chum Salmon Oncorhynchus keta.

[52] McCarthy JJ (2008). MicroRNA-206: the skeletal muscle-specific myomiR. Biochim Biophys Acta - Gene Regul Mech.

[53] Townley-tilson WHD, Callis T, Wang D (2010). MicroRNAs 1, 133 , and 206 : Critical factors of skeletal and cardiac muscle development, function, and disease. Int J BioChemiPhysics.

[54] Chen J-F, Mandel EM, Thomson JM, Wu Q, Callis TE, Hammond SM, Conlon FL, Wang D (2006). The role of microRNA-1 and microRNA-133 in skeletal muscle proliferation and differentiation. Nat Genet.

[55] Kern F, Ludwig N, Backes C, Maldener E, Fehlmann T, Suleymanov A, Meese E, Hecksteden A, Keller A, Meyer T (2019). Systematic assessment of blood-borne microRNAs highlights molecular profiles of endurance sport and carbohydrate uptake. Cells..

[56] Sokol NS, Ambros V (2005). Mesodermally expressed Drosophila microRNA-1 is regulated by Twist and is required in muscles during larval growth. Genes Dev.

[57] Zhao Y, Ransom JF, Li A, Vedantham V, von Drehle M, Muth AN, Tsuchihashi T, McManus MT, Schwartz RJ, Srivastava D (2007). Dysregulation of cardiogenesis, cardiac conduction, and cell cycle in mice lacking miRNA-1-2. Cell..

[58] Mishima Y, Abreu-Goodger C, Staton AA, Stahlhut C, Shou C, Cheng C, Gerstein M, Enright AJ, Giraldez AJ (2009). Zebrafish miR-1 and miR-133 shape muscle gene expression and regulate sarcomeric actin organization. Genes Dev.

[59] Christodoulou F, Raible F, Tomer R, Simakov O, Trachana K, Klaus S, Snyman H, Hannon GJ, Bork P, Arendt D (2010). Ancient animal microRNAs and the evolution of tissue identity. Nature..

[60] Lin CC, Chang YM, Pan CT, Chen CC, Ling L, Tsao KC, Yang RB, Li WH (2014). Functional evolution of cardiac microRNAs in heart development and functions. Mol Biol Evol.

[61] Horak M, Novak J, Bienertova-Vasku J (2016). Muscle-specific microRNAs in skeletal muscle development. Dev Biol.

[62] Ludwig N, Leidinger P, Becker K, Backes C, Fehlmann T, Pallasch C, Rheinheimer S, Meder B, Stähler C, Meese E, Keller A (2016). Distribution of miRNA expression across human tissues. Nucleic Acids Res.

[63] Yan B, Guo J-T, Zhao L, Zhao J (2012). microRNA expression signature in skeletal muscle of Nile tilapia. Aquaculture.

[64] Pineau P, Volinia S, McJunkin K, Marchio A, Battiston C, Terris B, Mazzaferro V, Lowe SW, Croce CM, Dejean A (2010). miR-221 overexpression contributes to liver tumorigenesis. Proc Natl Acad Sci U S A.

[65] Galardi S, Mercatelli N, Giorda E, Massalini S, Frajese GV, Ciafrè SA, Farace MG (2007). miR-221 and miR-222 expression affects the proliferation potential of human prostate carcinoma cell lines by targeting p27Kip1. J Biol Chem.

[66] Zhang XJ, Ye H, Zeng CW, He B, Zhang H, Chen YQ (2010). Dysregulation of miR-15a and miR-214 in human pancreatic cancer. J Hematol Oncol.

[67] Flynt AS, Li N, Thatcher EJ, Solnica-Krezel L, Patton JG (2007). Zebrafish miR-214 modulates Hedgehog signaling to specify muscle cell fate. Nat Genet.

[68] Yang H, Kong W, He L, Zhao JJ, O’Donnell JD, Wang J, Wenham RM, Coppola D, Kruk PA, Nicosia SV, Cheng JQ (2008). MicroRNA expression profiling in human ovarian cancer: miR-214 induces cell survival and cisplatin resistance by targeting PTEN. Cancer Res.

[69] Mao L, Liu S, Hu L, Jia L, Wang H, Guo M, Chen C, Liu Y, Xu L (2018). MiR-30 family: a promising regulator in development and disease. Biomed Res Int.

[70] Yuan B, Sun GJ, Zhang GL, Wu J, Xu C, Dai LS, Chen J, Yu XF, Zhao ZH, Zhang JB (2015). Identification of target genes for adenohypophysis-prefer miR-7 and miR-375 in cattle. Genet Mol Res.

[71] Baroukh NN, Van Obberghen E (2009). Function of microRNA-375 and microRNA-124a in pancreas and brain. FEBS J.

[72] Yu C, Li M, Wang Y, Liu Y, Yan C, Pan J, Liu J, Cui S, Cui S (2017). MiR-375 mediates CRH signaling pathway in inhibiting E2 synthesis in porcine ovary. Reproduction..

[73] Bizuayehu TT, Babiak J, Norberg B, Fernandes JMO, Johansen SD, Babiak I (2012). Sex-biased miRNA expression in atlantic halibut (Hippoglossus hippoglossus) brain and gonads. Sex Dev.

[74] Qiu W, Zhu Y, Wu Y, Yuan C, Chen K, Li M (2018). Identification and expression analysis of microRNAs in medaka gonads. Gene..

[75] Juanchich A, Le Cam A, Montfort J, Guiguen Y, Bobe J. Identification of differentially expressed miRNAs and their potential targets during fish ovarian development. Biol Reprod. 2013;88. 10.1095/biolreprod.112.105361. 10.1095/biolreprod.112.105361 23595902

[76] Bobe J, Labbé C (2010). Egg and sperm quality in fish. Gen Comp Endocrinol.

[77] Bromage N, Jones J, Randall C, Thrush M, Davies B, Springate J, Duston J, Barker G (1992). Broodstock management, fecundity, egg quality and the timing of egg production in the rainbow trout (Oncorhynchus mykiss). Aquaculture..

[78] Martin M (2011). Cutadapt removes adapter sequences from high-throughput sequencing reas. EMBnet J.

[79] Desvignes T, Loher P, Eilbeck K, Ma J, Urgese G, Fromm B, Sydes J, Aparicio-Puerta E, Barrera V, Espín R, Thibord F, Bofill-De Ros X, Londin E, Telonis AG, Ficarra E, Friedlä Nder MR, Postlethwait JH, Rigoutsos I, Hackenberg M, Vlachos IS, Halushka MK, Pantano L (2019). Unification of miRNA and isomiR research: the mirGFF3 format and the mirtop API. Bioinformatics..

[80] Galili T, O’Callaghan A, Sidi J, Sievert C (2018). Heatmaply: an R package for creating interactive cluster heatmaps for online publishing. Bioinformatics..

[81] Lê S, Josse J, Husson F (2008). FactoMineR: an R package for multivariate analysis. J Stat Softw.

[82] Love MI, Huber W, Anders S (2014). Moderated estimation of fold change and dispersion for RNA-seq data with DESeq2. Genome Biol.

[83] L. Pantano, Bioconductor - DEGreport: report of DEG analysis. R package version 1.26.0 http://lpantano.github.io/DEGreport/., 2020. https://bioconductor.org/packages/release/bioc/html/DEGreport.html (accessed February 4, 2021).

